# Cholecystokinin: Clinical aspects of the new biology

**DOI:** 10.1111/joim.20110

**Published:** 2025-06-25

**Authors:** Jens F. Rehfeld

**Affiliations:** ^1^ Department of Clinical Biochemistry Rigshospitalet University of Copenhagen Copenhagen Denmark

**Keywords:** cholecystokinin, cytokine, gut hormone, neurotransmitter, satiation factor, tumor‐marker

## Abstract

Cholecystokinin (CCK) is a classic gut hormone that has been known for almost a century to regulate gallbladder emptying, pancreatic enzyme secretion, and gastrointestinal motor activity. In 1968, the CCK structure was identified by Viktor Mutt and Erik Jorpes from porcine gut extracts as a peptide of 33 amino acid residues. Based on that structure, physiological, immunochemical, molecular, and cell biological research has since expanded the insight into the biology of CCK remarkably. Thus, CCK was the first identified intestinal satiety signal to the brain. Moreover, the CCK gene is now known to be expressed in different molecular forms not only in the gut, but very much so in central and peripheral neurons, in addition to extra‐intestinal endocrine cells, immune cells, cardiomyocytes, spermatogenic cells, and certain fat cells. Accordingly, CCK peptides function not only as hormones. They are also neurotransmitters, paracrine growth and satiation factors, anti‐inflammatory cytokines, incretins, adipokins, myokines, potential fertility factors, and tumor markers. Consequently, CCK biology has now opened windows for insights into pathophysiology with diagnostic and therapeutic possibilities in metabolic disorders (obesity, eating disorders, and diabetes mellitus), gallbladder disease, neuropsychiatric diseases (cerebral tumors, memory, and anxiety disorders), cardiac diseases (prognosis in heart failure), neuroendocrine and pediatric tumors, as well as perhaps infertility.

## Graphical abstract

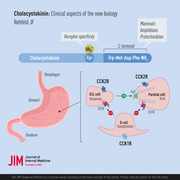

## Introduction

Endocrinology covers a wide range of different hormones: steroids, monoamines, proteins, and peptides. In modern peptide endocrinology, gastrointestinal hormones have recently surfaced as a strikingly dynamic area with considerable clinical impact [1]. It suffices to mention, for example, glucagon‐like peptide‐1 (GLP‐1) and glucose‐dependent insulinotropic polypeptide (GIP), from which several drugs have already been derived and used widely in the treatment of diabetes and obesity, with additional candidates in the pipeline and potential for therapy of cardiovascular, renal, and cerebral diseases as well [2, 3, 4].

GIP and GLP‐1 are well described as polypeptides secreted from intestinal K‐ and L‐cells, respectively [1]. Other gastrointestinal hormones, however, have a more complex biology. Among these is not least cholecystokinin (CCK), whose role as a hormone in digestion may turn out to be secondary compared to some of the extraintestinal activities implicated in the new biology.

Following a summary of the CCK history, this article will offer a brief overview of the new CCK biology, including phylogeny, biogenesis, cellular expression with cell‐specific peptide patterns, receptors, and subsequently derived functions. A note on reliability of CCK measurements will also be given before describing the perspective of pathophysiological involvement of CCK in metabolic disorders, gallbladder pathology, neuropsychiatric diseases, cardiac diseases, and various neoplasias.

## A short history

**Fig. 1:**
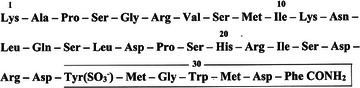
The legacy of Viktor Mutt, that is, the structure of the 33 amino acid residues of porcine cholecystokinin as published by Mutt and Jorpes in 1968 (see ref. [12]). Note that the tyrosyl residue in position 27 is O‐sulfated and that the phenylalanyl residue in position 33 is carboxyamidated. The C‐terminal boxed sequence is the bioactive, receptor‐bound epitope of CCK.

More than a century ago, some European physiology laboratories observed that administration of acid into the canine duodenum stimulated bile secretion from the liver and gallbladder contraction [5, 6, 7, 8; for review, see ref. 9]. For decades, the question was whether the effect was due to the first discovered gut hormone, secretin [10], or to a yet unknown hormone. In 1928, however, Ivy and Oldberg provided evidence that the gallbladder effect could not be ascribed to secretin. Consequently, implication of a new gut hormone was necessary to explain the activity. Ivy and Oldberg named the hormone CCK [11]. Subsequently, a few laboratories tried to purify CCK from the small intestine—in vain, however, because the necessary biochemical purification technologies were not yet available. Therefore, not until Viktor Mutt and Erik Jorpes in Stockholm in the 1950s and 1960s established a large plant for extraction and purification from vast amounts of porcine jejunal mucosa (20 km small intestine) did structure identification become possible. Hence, Mutt and Jorpes identified a peptide with CCK activity. The structure was published in 1968 [12]. It had a sequence of 33 amino acid residues, a carboxyamidated C‐terminal sequence homologous to that of gastrin, and besides, it was tyrosyl *O*‐sulfated (Fig. 1). Elucidating the structure of this peptide became a decisive milestone. With in vitro synthesized fragments of CCK‐33, plenty of material became available for physiological studies, for production of antibodies to be used in immunoassays and immunohistochemistry, as well as for identification of the CCK gene and new molecular forms of CCK peptides. These tools together with other methods from molecular and cell biology paved the way for a number of paradigmatic shifts that have unfolded a surprisingly multifaceted biology of CCK. Consequently, CCK is now seen as a ubiquitous peptide messenger system in the body [13]. The basic chemistry and biology has recently been detailed elsewhere [14]. Below follows a summary of these aspects as premises for the discussion of clinical perspectives. First, however, two useful definitions are presented.

## Definitions

### The CCK system

The system comprises the specific molecular elements in the biogenesis and function of CCK. Thus, gene, mRNA, biosynthetic precursors (prepro‐ and proCCK), processing intermediates, and mature bioactive peptides (CCK‐83, ‐58, ‐33, ‐22, ‐8, and ‐5), as well as receptors (CCK_1_, CCK_2_, and CCK_3_), constitute the CCK system.

### The biology of CCK

At first, the biology was simple: CCK was a single peptide (CCK‐33) released from a specific endocrine gut cell to regulate gallbladder contraction, somewhat in the same way as insulin and glucagon are singular peptides released from specific pancreatic islet cells to regulate metabolism. The *new* biology, however, is the sum of additional evolutionary, biochemical, and physiological insights obtained since Mutt and Jorpes identified CCK‐33 in gut extracts [12]. Several of the new insights—for instance, cerebral neurotransmitter effects, gut‐brain satiety effects, and anti‐inflammatory cytokine effects—may almost overshadow the significance of the original concept of CCK as just a regulator of gallbladder contraction [14].

## The new biology of CCK

### Phylogenesis

**Fig. 2:**
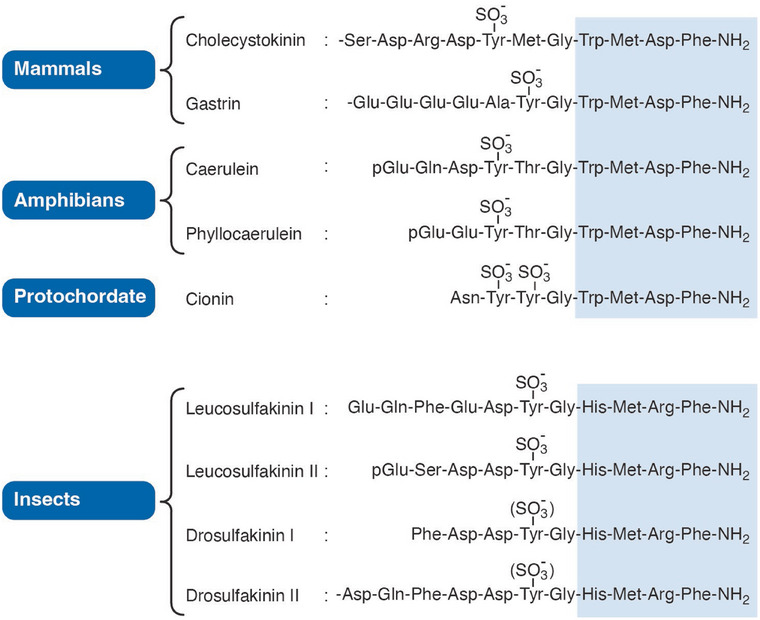
The decisive homologous structure in members of the cholecystokinin peptide family in mammals, amphibians, protochordates (Ciona intestinalis), and insects (a cockroach (Leucophaea madera) and a fruit fly (Drosophila melanogaster). Note that the boxed C‐terminal tetrapeptide sequences are all carboxyamidated and that this tetrapeptide amide as such is the core structure necessary for receptor binding. Note also O‐sulfation of the more N‐terminal tyrosyl residues. The tyrosyl‐sulfation is a derivatization that modifies the affinity and hence the specificity of receptor bindings.

CCK is a member of a family that in mammals includes the antral hormone *gastrin* [15]; in amphibians, the skin peptide *caerulein* [16, 17]; and in protochordates, the neuropeptide *cionin* [18]. Insect neuropeptides, the *sulfokinins* [19, 20], are also structurally related and therefore included in the family (Fig. 2). Evolutionary studies of the genes indicate that CCK peptides originated almost 600 million years ago [21, 22, 23, 24].

### Biogenesis and molecular heterogeneity of peptides

There is one CCK gene [25, 26], which is transcribed to a single mRNA of 750 bases [25, 26, 27]. The mRNA molecule is then translated to a precursor protein (preproCCK) with a sequence of 115 amino acid residues [27]. The N‐terminal signal or pre‐sequence is probably removed during translation, leaving a proCCK of 95 amino acids. ProCCK subsequently undergoes extensive posttranslational modifications by a multitude of processing enzymes acting along cellular secretory or synaptic pathways [28, 29, 30, 31, 32, 33, 34, 35, 36, 37]. The covalent modifications comprise endoproteolytic cleavages at mono‐ and dibasic sites, amino acid derivatizations, and N‐ or C‐terminal trimmings. Notably, none of the modifications are complete. As a result of the posttranslational processing, a number of different bioactive CCK peptides are accumulated in the secretory granules (endocrine cells) or presynaptic vesicles (neurons): CCK‐83, ‐58, ‐33, ‐22, ‐8, and ‐5. The first five occur in both *O*‐sulfated and nonsulfated forms, whereas CCK‐5 (without a tyrosyl residue) is unsulfated. The peptide patterns, however, are cell‐specific (for recent reviews, see refs. [14, 37]).

### The widespread tissue and cell expression

In accordance with the classical role as gut hormone, specific endocrine cells in the intestinal mucosa, named I‐cells, have been identified in the duodenum and proximal jejunum as the major source of intestinal CCK peptides [38, 39, 40]. Presumably, a majority of CCK in plasma originates from these cells. The I‐cells, however, are present also in the distal jejunum, in the entire ileum, and sporadically in the mucosa of the proximal colon [41, 42]. Because the duodenum is very short in comparison with the remaining small intestine, jejunum as well as ileum each produce considerably more CCK than the duodenum [41, 42, 43 and unpublished results]. Thus, it is misleading to characterize CCK primarily as a duodenal hormone, as still seen in several textbooks.

Most CCK in mammals is, however, not synthesized in the gut but in neurons, central as well as peripheral [30, 37, 44, 45, 46, 47, 48]. Estimated by concentrations of CCK mRNA, the brain expresses four–five times more CCK than the small intestinal mucosa (unpublished results). The cerebral CCK neurons also express “classic” transmitters, such as GABA in interneurons and glutamate in pyramidal and thalamic neurons [48]. Moreover, the gut and pancreatic islets contain CCK neurons, especially abundant in the colon [44, 49].

Although cerebral neurons and intestinal I‐cells are clearly the two major expression sites, specific expression has in addition been detected in other cells (Table 1). They include pituitary corticotrophs [50, 51], thyroid C‐cells [52, 53], adrenal medullary cells [54], spermatogenic cells [55, 56], cardiac myocytes [57], circulating monocytes [58, 59, 60], and fat cells, at least in some mammalian species (unpublished results).

**Table 1:** Tissue concentrations of bioactive cholecystokinin (CCK) peptides in mammals.

Tissue	Tissue content^a^ (pmol/g)
Intestinal Tract:	
Duodenal mucosa	200
Jejunal mucosa	150
Ileal mucosa	20
Colonic mucosa	5
Central Nervous System:	
Cerebral cortex	400
Hippocampus	350
Hypothalamus	200
Cerebellum	2
Spinal cord	40
Peripheral Nervous System:	
Vagal nerve	25
Sciatic nerve	15
Nerveplexes in colonic wall	5
Extraintestinal Endocrine Glands:	
Adenohypophysis	25
Neurohypophysis	20
Thyroid gland	2
Adrenal medulla	1
Urogenital Tract:	
Testicles	5
Spermatozoas	1
Cardiovascular System:	
Atrial myocytes	10
Ventricular myocytes	2
Mononuclear Immune Cells ^b^:	++

^a^ Orders of magnitude based on measurement of tissue extracts from several mammalian species.

^b^ Expression determined by immunocytochemistry of monocytes.

### Cell‐specificity of CCK peptide expression

As shown in Table 2, there are at least three different molecular patterns of bioactive peptides in cells expressing the CCK gene. *First*, in both central and peripheral neurons, *O*‐sulfated CCK‐8 and the unsulfated CCK‐5 predominate. A similar pattern is seen in adrenal medullary cells (which are neuronally derived). *Second*, gut endocrine I‐cells express a considerably broader pattern that includes large molecular forms (CCK‐58, ‐33, and ‐22) as well as short forms (CCK‐8 and ‐5) [61], where CCK‐33 is the predominant form in humans [62], but CCK‐58 in dogs [63]. The four longest CCK peptides in I‐cells all occur in sulfated and nonsulfated variants [35]. Finally, unique and specific patterns are found in lower concentrations in pituitary corticotrophs, thyroid C‐cells, spermatogenic cells, cardiac myocytes, and circulating monocytes [50, 51, 52, 53, 54, 55, 56, 57, 58, 59].

**Table 2:** Cell‐specific patterns in mammals of predominant cholecystokinin (CCK) peptides.

Cells	Peptide pattern
Intestinal endocrine cells (I‐cells)	CCK‐58, ‐33, ‐22, ‐8, and ‐5 (s and ns)
Cerebral neurons	CCK‐8 (s) and CCK‐5
Peripheral neurons	CCK‐8 (s) and CCK‐5
Pituitary corticotrophs	CCK‐83 (s), CCK‐58 (s), and CCK‐33 (s)
Thyroid C‐cells	CCK‐8 (ns) and CCK‐5
Adrenal medullary cells	CCK‐8 (ns and s)
Spermatozoas	CCK‐22 (ns and s) and CCK‐8 (ns)
Cardiomyocytes	proCCK (25‐94) and CCK‐8 (s)

### CCK receptors

**Fig. 3:**
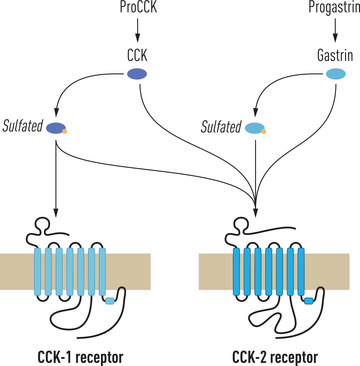
The homologous biosynthetic precursors, proCCK and progastrin, are both processed to O‐sulfated (s) as well as non‐sulfated (ns) peptide agonists for the cholecystokinin (CCK) receptors. Only sulfated CCK peptides are bound with high affinity to the CCK_1_ receptor, whereas the promiscuous CCK_2_ receptor binds all carboxyamidated products of both proCCK and progastrin.

Using isotope‐labeled CCK fragments or analogues as agonists as well as synthetic antagonists, the distribution and distinction between “alimentary” (CCK_A_) and “brain” (CCK_B_) receptors were studied already in the 1970s and 1980s [64, 65]. In 1992, however, the structures of both the CCK_A_ and the gastrin/CCK_B_ receptors were deduced after cDNA cloning [66, 67]. The receptors are now named CCK_1_ and CCK_2_, respectively. Later, informative reviews of receptor distribution, physiology, and pharmacology have been published [see refs 68, 69]. Very recently, however, a third CCK receptor (GPR 173) has also been identified in cerebral tissue [70]. Further information about expression of this CCK_3_ receptor also in extracerebral tissue remains to be published. The binding specificity of the CCK_1_ and CCK_2_ receptors differs. Thus, all carboxyamidated CCK and gastrin peptides, irrespective of sulfation, are bound with similar affinities to the gastrin/CCK_2_ receptor, whereas the CCK_1_ receptor is more selective and binds only tyrosyl *O*‐sulfated CCK peptides (Fig. 3).

### CCK functions

An overview of the multiple functions of the CCK system requires recognition of the new biology in its entirety: On one hand, there are the different cellular origins and release mechanisms for bioactive CCK peptides. On the other hand, there are the targets in terms of CCK receptors and the routes by which the peptides travel to reach the receptors on the target cells (the endocrine, neurocrine, paracrine, autocrine, spermiocrine secretory ways, and synaptic transmissions). The CCK receptors are expressed in a majority of organs and tissues in the body: central and peripheral neurons as well as glial cells; the entire gastrointestinal tract; myocytes of the gallbladder and the sphincter Oddi muscles; exocrine and endocrine pancreatic cells; the cardiovascular system; the urogenital system; and the immune system [68, 69]. Consequently, CCK peptides have many labels and may act independently as neurotransmitters, hormones, growth factors, satiation factors, cytokines, myokines, and perhaps adipokines and fertility factors.

**Fig. 4:**
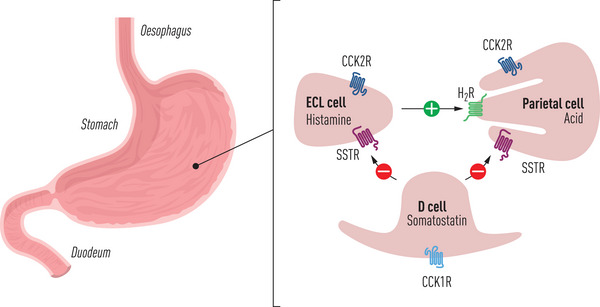
Cholecystokinin (CCK) influences gastric acid secretion: CCK peptides inhibit acid secretion in normal healthy subjects and animals after binding of blood‐borne sulfated CCK to the CCK_1_ receptor on gastric D‐cells. Subsequently, the D‐cell release via somatostatin to the somatostatin_2_ receptor on parietal cells blocks further acid secretion. Although both sulfated and non‐sulfated CCK peptides also are bound to the CCK_2_ receptor on ECL and parietal cells, their acid stimulatory effect is marginal in comparison with that of gastrin that circulates in 10‐fold higher concentration in healthy subjects. In CCKoma patients (with massive overproduction of non‐sulfated CCK peptides (refs. [176, 177]), the effect is opposite, that is, hypersecretion of gastric acid and development of duodenal ulcers. The acid stimulatory effect in these patients occurs because the CCK_1_ receptor cannot bind non‐sulfated CCK peptides, and thus the D‐cell does release only limited amounts of the inhibitory somatostatin. In other words, non‐sulfated CCK peptides act like gastrin peptides.

On top of this picture, it is of fundamental significance to realize that the target cells for CCK peptides also express receptors for a multitude of other bioactive peptide systems. Therefore, different peptide systems interact and cross‐talk along intracellular signal‐transduction pathways. Well‐known examples are the mutual potentiation of CCK peptides with secretin in their stimulation of exocrine pancreatic enzyme and bicarbonate secretion [71, 72]; the CCK/gastrin potentiation of GLP‐1 in stimulation of pancreatic beta‐cell growth [73]; the interaction of CCK with GLP‐1 and PYY in signaling to the brain via afferent vagal fibers to regulate food intake [74]; and, for instance, the balance between sulfated and nonsulfated CCK peptides from CCKomas in the inhibition or stimulation of gastric acid secretion [75] (Fig. 4). It is likely that studies of the interactive physiology between CCK and other bioactive peptide systems, as well as the interaction with non‐peptidergic extracellular messenger molecules, will be an essential part of the future physiology and pharmacology of CCK [76].

## Measurement of CCK

It is obvious that progress in understanding the basic CCK biology as well as the roles of CCK in pathophysiology, diagnostics, and therapy has been and henceforth will have to be based on reliable measurements. So far, such reliability has been a problem. Therefore, a comment about the premises for accuracy of CCK assays and about the methods required to obtain the desired accuracy:

### Assay premises

CCK is a most, if not the most, difficult peptide system to measure accurately in biological fluids, particularly so in plasma. The difficulty is caused by the biochemistry of CCK: first, the molecular heterogeneity with different peptides (CCK‐58, ‐33, ‐22, and ‐8) requires assays that measure these forms with equimolar potency. Second, the concentrations of CCK peptides in circulation are very low for a hormone. In mammals, the concentrations in fasting are often femtomolar. After meals, they increase to a few picomolar levels (3–5 pM). Measurement of these levels require high‐affinity antibodies in the immunoassays. Third, the related sister hormone, gastrin (Fig. 2), circulates in 10‐fold higher concentrations in plasma. Consequently, far most antibodies raised for immunoassays against CCK will cross‐react with gastrin peptides. Finally, the high protein concentrations in plasma may interfere unspecifically with immunoassay measurements. Therefore, preanalytical extraction from protein‐rich fluids is necessary.

### Assay methods

There are three relevant methodologies to consider for CCK measurements in biological fluids (blood plasma, cerebrospinal fluid, semen, and tissue extracts). *Bioassays* have historically been crucial for the initial purifications from intestinal tissue and, for instance, amphibian skin tissue extracts [12, 16, 77]. However, in general, bioassays suffer from insufficient specificity and sensitivity for plasma measurements. One exception has nevertheless been reported [78]. It is based on amylase secretion from isolated pancreatic cells and is sufficiently sensitive and specific for plasma measurements. However, in spite of the impressive reliability, it has been too complex, labor intensive, and costly for practical purposes and hence for wider applicability. An alternative principle of sufficient sensitivity and practicability is the *immunoassay* technology, especially radioimmunoassay methods (RIA). Establishment of RIAs of sufficient reliability for CCK has, however, as described above, also been a challenge. This is abundantly illustrated by many unspecific and hence misleading commercial CCK immunoassay kits that have been and still are on the market [79, 80]. Nevertheless, a few specific antisera for CCK RIAs have been raised in university laboratories. These RIAs do not cross‐react with gastrin (neither in sulfated nor non‐sulfated forms of gastrin) [79, 81, 82, 83, 84, 85, 86]. Moreover, most of these seem to measure the different molecular forms of bioactive CCK peptides in plasma and tissue extracts adequately [79, 86]. The *third* assay principle to consider is *mass spectrometry* (MS). For measurement of bioactive CCK peptides that in the basal conditions circulate in plasma in femtomolar concentrations, MS methods have, however, so far been useless due to insufficient analytical sensitivity. In addition, they are also costly. Nevertheless, an MS‐based method using statistical modeling software was recently reported to be able to measure an N‐terminal fragment of proCCK as a surrogate parameter of CCK secretion into plasma in human subjects [87]. This is possible because proCCK and some of its intermediate processing fragments are known to circulate in substantially higher concentrations than the bioactive CCK peptides [88, 89]. Thus, further improvements in MS technology may be relevant to follow. Additional details in the measurement of CCK is discussed elsewhere [90, 91].

Progress in knowledge about the role of CCK in diseases in general has been slow in comparison with that of most other peptide hormones because of the limitations in the availability of assays that measure the true concentrations of CCK [79, 86]. As a conclusion of the assay discussion, however, the recommendation of today will be to use an entirely specific RIA with an antiserum having characteristics similar to the one detailed in ref. [86].

## Pathophysiology of CCK

### CCK in obesity and eating disorders

CCK was the first discovered intestinal satiety signal and is by now well‐established as such [92]. It reaches the brain via CCK_1_ receptors expressed on afferent vagal fibers [74, 93, 94, 95]. Consequently, there has been an impressive interest in measuring intestinal CCK secretion in human obesity before as well as after various treatments (diets, exercise, gastric bypass, and sleeve surgery) [96, 97, 98, 99, 100, 101, 102, 103, 104, 105, 106, 107, 108, 109, 110, 111, 112, 113, 114]. Moreover, intestinal CCK secretion has also been examined in patients suffering from eating disorders (anorexia nervosa, anorexia in the aging, and bulimia nervosa) [115, 116, 117, 118, 119, 120, 121, 122, 123, 124, 125, 126, 127].

The obesity studies showed that the plasma CCK concentrations both in the fasting state and after intake of mixed meals are the same in lean and obese people. Neither weight nor sex apparently influences the secretion of CCK [105]. Diet plus exercise‐induced weight loss in obese patients maintained over 2 years may increase CCK secretion both in the basal state and after meals, but only slightly [108]. Weight loss in severely obese patients after gastric bypass operations, however, influences the secretion of CCK. Hence, the basal secretion in the fasting state decreases to concentrations from 1 pmol/L before to 0.35 pmol/L after the operation. After ingestion of mixed meals, however, the peak concentrations increase from 3–5 pmol/L preoperatively to 7–8 pmol/L postoperatively [101, 103, 106, 111]. Hence, in unoperated obese patients, as in lean subjects, there is a five‐fold increase in postprandial CCK concentration. In contrast, the postprandial CCK concentrations increase by 20‐ to 25‐fold in bypass‐operated patients. Such difference renders it likely that meal‐induced CCK secretion contributes to the increased postoperative incretin effect, which in 80% of the bypassed patients reduces or eliminates type 2 diabetes mellitus.

In anorexia nervosa, the CCK results are ambiguous, which to some extent may be explained by assay differences and small numbers of the mainly young girls who have been studied [116, 117, 118, 119, 120]. Generally, there seems to be an increased CCK secretion in the young anorexia patients. The increase, however, is hardly causal but might interfere with refeeding and recovery [128].

Anorexia is quite common among older people. It has been estimated to vary between 15% and 30% and is even higher in nursing homes [121]. Both fasting and food‐stimulated CCK secretion seems to increase with age [122]. Moreover, the effect of exogenous CCK on food intake also appears increased in aging [123], indicating that CCK may play a role, although hardly a major one [128].

CCK secretion in bulimia nervosa has also been examined [124, 125, 126, 127]. Some studies found decreased basal and postprandial CCK concentrations in plasma but also slower gastric emptying. It is therefore possible that the decreased plasma levels to some extent are due to delayed gastric emptying with subsequently reduced food stimulation of the CCK cells in the proximal gut.

In conclusion, the changes in CCK secretion and effects found in obesity and eating disorders are modest and as such hardly decisive. However, it is possible that the significance of even minor variations should be sought in the interaction/potentiation of CCK peptides with other peptide messengers such as GLP‐1 and PYY, as suggested elsewhere [76].

### CCK in diabetes mellitus

CCK peptides have evoked some interest in diabetes research, not least because CCK, in itself being a minor incretin, may potentiate the effect of major incretins such as GLP‐1 and GIP. Details of this area are described in recent reviews [129, 130]. Regarding intestinal CCK secretion in diabetes, a reduced postprandial CCK response to low‐fat meals has been observed in patients with type 2 diabetes [131, 132]. The responses to medium‐ and high‐fat meals, however, are normal [132]. Moreover, in accordance with the intestinal mucosal CCK concentrations and I‐cell densities in patients with type 2 diabetes, these parameters are also normal [43]. In type 1 diabetes, abnormal intestinal CCK release has so far not been encountered. Thus, the intestinal CCK synthesis and secretion are apparently not changed to any significant extent in diabetes.

The major premise for associating CCK with diabetes is, nevertheless, the expression of CCK receptors and peptides as well as the sister hormone gastrin in pancreatic islets. Such expression has also been found in experimental diabetes in rodents as well as in human diabetes [73, 133, 134, 135, 136, 137, 138, 139, 140, 141]. On this background, promising anti‐diabetic effects of some modified CCK‐8 analogues (not least [pGlu‐Gln] CCK‐8) have been demonstrated [73, 142, 143, 144, 145]. However, for attempts to treat diabetes in humans with CCK receptor agonists, for safety reasons [146], it has to be low doses of the agonist in combination with analogues of the major incretins such as GLP‐1 and GIP (for review, see ref. [129]).

### CCK, gallbladder, and exocrine pancreatic diseases

The association between CCK and gallbladder function is, per definition, the basis for the century‐old biology of CCK, as reflected also in the name of the hormone [11]. Today it is an established fact that CCK peptides from I‐cells in the proximal small intestine in a concentration‐related manner control the intestinal phase of gallbladder emptying via CCK_1_ receptors expressed on gallbladder muscles (for recent review, see ref. [147]). The mechanistic details and the relationship to gastrointestinal disorders, pancreatic insufficiency, and gallstone/gallbladder diseases have systematically been studied, not least by Masclee and Lamers et al. [147, 148, 149, 150, 151, 152, 153].

The results are summarized in Table 3, which shows that the occurrence of gallstones frequently accompanies the examined disorders. Although gallstone attacks are painful and annoying, surgical gallbladder removal is today fast and uncomplicated, and life without a gallbladder is pretty normal. Remember that the successful rodent, the rat, does not have a gallbladder. Thus, perhaps the pancreozymic effect of CCK should be considered more essential for digestion than the gallbladder‐emptying effect, as demonstrated by normal rats.

**Table 3:** Cholecystokinin secretion and gallbladder motility in some gastrointestinal disorders.^a^

Disorder	Stimulus	CCK secretion	Gallbladder motility	Gallstone prevalence	Mechanism
Partial gastrectomy	Oral meal	Increased	Increased	Increased	Rapid gastric emptying
Sleeve gastrectomy	Oral meal	Increased	Normal	–	Rapid gastric emptying
Roux‐en‐Y bypass	Oral meal	Increased	Decreased	Increased	Rapid gastric emptying with bypass of prox. gut
Celiac disease	Oral meal	Decreased	Decreased	Increased	–
Exocrine pancreatic insufficiency	Oral meal	Decreased	Decreased	Increased	Delayed intestinal fat digestion
Whipple operation	Oral meal	Decreased	Gallbladder removed	–	Reduced pancreatic enzyme secretion
Colectomy	Oral meal	Increased	Normal	Increased	Changes in bile composition
Parenteral nutrition	No enteral feeding	Decreased	Stasis	Increased	No enteral stimulus

^a^ See also ref. [147] for further details.

### CCK in neuropsychiatric diseases

As already mentioned, the majority of CCK peptides in mammals, including humans, are synthesized as neurotransmitters in neurons [30, 31, 32, 47]. Thus, the cerebral regions (except the cerebellum) are loaded with CCK neurons, short interneurons as well as long‐reaching neurons [30, 44, 45, 46, 47, 48]. Considering the hundreds or thousands of contacts each neuron has with other neurons, glial cells, blood vessels, and so on, it looks like an almost insurmountable challenge to identify and rule out the CCK circuits in the brain and how they are involved in the pathophysiology of neuropsychiatric diseases.

Some superficial hints, however, have been given by comparing CCK concentrations in cerebrospinal fluid from patients and healthy controls. Thus, in patients with depression, schizophrenia, Parkinson's disease, and severe alcohol dependence, the cerebrospinal CCK concentrations are decreased but still overlapping with normal levels [154, 155, 156, 157]. In contrast, the CCK levels are increased in multiple sclerosis [158]. Moreover, CCK‐knockout (KO) mice contribute to information about the role of cerebral CCK. Hence, global KO of the CCK gene shows that mice have lost their memory and exhibit increased anxiety [159, 160], in addition to peripheral disturbances of digestive and endocrine functions [159, 160, 161, 162] that also may interfere with memory functions [163, 164]. The increased anxiety in the CCK‐KO mice is paradoxical, because it is now well established that peripheral administration of exogenous CCK‐4 and CCK‐8 in a dose‐related manner induces panic attacks of anxiety in mammals, among which human subjects have been carefully studied [165, 166, 167]. A further player in cerebral anxiety mechanisms is obviously the CCK_2_ receptor. Thus, KO mice without this receptor show less anxious behavior in comparison with wild‐type mice [168]. On top of the role of CCK in anxiety and panic disorders, it has now been suggested that CCK peptides may be promising drug candidates for the therapy of Alzheimer's and Parkinson's diseases [169]. Finally, it also deserves mention that CCK in dopaminergic mesolimbic neurons projecting to the frontal brain might be of relevance for the pathophysiology of schizophrenia [170].

### CCK in tumors

Like other gut hormones, bioactive CCK peptides are also growth factors [171, 172]. Furthermore, because CCK to a variable extent is expressed in neuroendocrine tumors and may function as tumor marker, a short presentation of the oncogenetic occurrence of CCK may have clinical interest (for reviews, see refs. [173, 174]). Of new and particular clinical interest is the rare pancreatic islet‐cell carcinoma with massive hyperCCKemia that causes a specific CCKoma syndrome [75, 175, 176, 177]. The major symptoms of the syndrome are severe non‐watery diarrhea, weight loss, recurrent peptic ulcers, and gallbladder disease with repeated gallstone attacks [176, 177]. The duodenal ulcer disease and diarrhea in spite of permanently low gastrin concentrations in plasma suggest that some CCK peptides from the pancreatic CCKoma and its metastases may induce gastrinoma‐like symptoms, that is, Zollinger–Ellison syndrome [75]. However, sulfated CCK peptides *inhibit* gastric acid secretion via CCK_1_ receptors on fundic somatostatin cells (Fig. 4). In contrast, non‐sulfated CCK peptides *stimulate* gastric acid secretion via CCK_2_‐receptors on gastric ECL cells. In other words, non‐sulfated CCK acts like gastrin. CCKoma cells may release manyfold more nonsulfated than sulfated CCK peptides [75, 177]. In addition, CCK is also expressed at lower levels in pituitary Cushing and Nelson tumors [178], in thyroid C‐cell carcinomas [53], and in pheochromocytomas [54]. Moreover, increased proCCK expression has been encountered in pediatric round cell Askin tumors [89], neuroepitheliomas, rhabdomyocarcomas, and Ewing sarcomas [179], where in the latter plasma proCCK measurements have turned out to be a promising marker [180]. In the brain, acoustic neuromas [181], astrocytomas [182], and gliomas [183, 184] synthesize CCK peptide and express CCK receptors. So far, however, proper hyperCCKemia in humans has been recorded only in association with the mentioned metastatic pancreatic CCKoma [75, 176, 177].

It is always a question whether peptide hormones expressed in cell lines also mirror expression in the original tumors from which the cell lines are derived. Moreover, the amount and molecular form of the hormone as released in vivo from the original tumor also matters in terms of clinical phenotype. Nevertheless, beyond the transplantable pancreatic islet cell line [175], CCK gene expression at the peptide level has been encountered also in a human bronchial small‐cell carcinoma cell line [185] and a medullary thyroid carcinoma cell line [186].

## Clinical perspectives: a summary

Considering the many different diseases in which CCK seems to be involved, much clinical research remains to be performed in order to establish the roles of CCK in pathophysiology, in diagnosis, and in therapy. The diseases mentioned in the present review are enumerated in Table 4. However, the research should always be carried out in awareness about the premise that the action of CCK on their target cells often will be a potentiating interaction with other messenger systems. Among such other systems, several will be other hormonal gastrointestinal peptides, other neuropeptide transmitters, or sometimes other cytokine peptides. That is complicated, but complexity is an inborn feature of the new biology.

**Table 4:** Cholecystokinin in diseases.

Disorder	Role of CCK	Degree of evidence
Metabolic disorders		
Obesity before bypass	None	+++
Obesity after bypass	↑ Incretin effect	++
Diabetes type 1	Exogenous CCK	–
Diabetes type 2	For incretin potentiation	++
Anorexia nervosa	None	+
Bulimia nervosa	None	+
Gastrointestinal disorders		
Partial gastrectomy	↑ Secretion	+++
Sleeve gastrectomy	↑ Secretion	+++
Gastric bypass	↑ Secretion	+++
Colectomy	↑ Secretion	+++
Celiac disease	↓ Secretion	++
Pancreatic insufficiency	↓ Secretion	++
Fructose intolerance	↑ Secretion	+
Neuropsychiatric disorders		
Depression	↓ CSF levels	+
Schizophrenia	↓ CSF levels	+
Parkinson's disease	↓ CSF levels	+
Multiple sclerosis	↑ CSF levels	+
Panic anxiety	Exogenous CCK‐4 provokes attacks	+++
Alzheimer's disease	Potential drug candidate	
Cardiovascular disorders		
Heart failure	ProCCK as prognostic marker	++
Neuroendocrine tumors		
Pancreatic CCKoma	Diagnostic marker	++
Cushing tumors	Diagnostic marker	+
Nelson tumors	Diagnostic marker	+
Thyroid C‐cell cancer	Diagnostic marker	+
Pheochromocytomas	Diagnostic marker	+
Pediatric tumors		
Askin tumors	Diagnostic marker	+
Neuroepitheliomas	Diagnostic marker	+
Rhabdomyosarcomas	Diagnostic marker	+
Ewing sarcomas	Diagnostic marker	++

With this background, some examples of obvious clinical applications can be proposed: In *pharmacotherapy*, new combination drugs should be developed. They should be based on a combination of CCK peptides (for instance, the CCK_2_ receptor agonist nonsulfated CCK‐5) with other gut hormones such as GLP‐1 and perhaps GIP. The fraction of CCK‐5 should be sufficiently low to avoid damage in pancreatic exocrine cells, whereas CCK_2_ receptor endocrine cells and neurons in the pancreas and the brain, respectively, could be activated and potentiate the already known effects of GLP‐1. Such drug(s) might improve therapy in both metabolic (obesity and type 2 diabetes mellitus [2]) and cerebral Alzheimer's and Parkinson's diseases.

In *diagnostic oncology*, it is described above that measurements of CCK or proCCK are useful markers for CCKomas and Ewing sarcomas. CCKoma is apparently a very rare neuroendocrine tumor. However, in order to ensure its prevalence and to learn more about the symptoms, larger series of plasma from neuroendocrine tumor cohorts in general and Zollinger–Ellison patients in particular [75] should be screened with reliable CCK assays as explained, discussed, and recommended in section V of this article (measurement of CCK). Furthermore, also plasma from patients with small‐cell lung carcinomas and thyroid cancer should be screened, because CCK peptides are expressed in cell lines from these tumors [185, 186].

In *cardiology*, there is still only little knowledge about the pathobiochemical and pathophysiological role of the proCCK fragments expressed in the cardial myocytes. The initial study of CCK in the heart [57] revealed that measurement of the cardiac proCCK fragment in plasma could be a prognostic risk factor for cardiac mortality. But there is a need for considerably more information, also about CCK receptor expression in the heart [187, 188].

In *endocrinology*, still nothing is known about plasma CCK levels in patients with pituitary corticotrophic tumors [178], thyroid C‐cell tumors [53], and pheochromocytomas [54]. Because these tumors express both bioactive CCK and CCK precursors, it is still an open question whether CCK peptides influence the pathophysiology and whether plasma CCK measurements might have diagnostic and/or prognostic significance.

Finally, in *gastroenterology*, the new recognition about CCK synthesis in the lower jejunum, the entire ileum, and also occasionally in the proximal colon [42] makes it worth examining the pre‐ and postprandial secretion of CCK in small intestinal diseases. For instance, the effects of fructose intolerance on CCK secretion might also be worth pursuing [189].

## Author contributions

**Jens F. Rehfeld**: Writing—original draft; conceptualization; investigation; methodology; validation; visualization; writing—review and editing; software; formal analysis; project administration; data curation; supervision; resources.

## Conflict of interest statement

The author declares no conflicts of interest.

## Funding information

The writing of this article received no funding. Earlier cholecystokinin studies in the laboratory of the author, however, received support from the Danish Medical Research Council, the Danish Cancer Union, and the Novo Nordic Foundation.

## Data Availability

Data sharing is not relevant for the present perspective article.

## References

[1] Rehfeld JF , Goetze JP . Gastrointestinal hormones: history, biology, and measurement. Adv Clin Chem. 2024;118:111–154. 38280804 10.1016/bs.acc.2023.11.004

[2] Madsbad S , Holst JJ . The promise of glucagon‐like peptide 1 receptor agonists (GLP‐1RA) for the treatment of obesity: a look at phase 2 and 3 pipelines. Expert Opin Investig Drugs. 2025;34(3):197–215. 10.1080/13543784.2025.2472408 40022548

[3] Marso SP , Daniels GH , Brown‐Frandsen K , Kristensen P , Mann JFE , Nauck MA , et al. Liraglutide and cardiovascular outcomes in type 2 diabetes. N Engl J Med. 2016;375:311–322. 27295427 10.1056/NEJMoa1603827 PMC4985288

[4] Jastreboff AM , Aronne LJ , Ahmad NN , Wharton S , Connery L , Alves B , et al. Tirzepatide once weekly for the treatment of obesity. N Engl J Med. 2022;387:205–216. 35658024 10.1056/NEJMoa2206038

[5] Bernard C . Mémoire sur le pancreas. Compte Rend Acad Sci. 1856;55 (suppl 1):379–563.

[6] Wertheimer E . De l'action des acides et du choral sur la secretion biliaire. Compte Rend Soc Biol. 1903;55:286–287.

[7] Fleig C . Réflex de l'acide sur la secretion biliaire. Compte Rend Soc Biol. 1903;55:353–355.

[8] Okada S . On the secretion of bile. J Physiol. 1914;49:457–482. 10.1113/jphysiol.1915.sp001722 PMC1420532 16993308

[9] Rehfeld JF , Feinle‐Bisset C . Milestones in the history of CCK. In: Feinle‐Bisset C , Rehfeld JF , editors. *Cholecystokinin*—*from gallbladder to cognition and beyond* . Elsevier/Academic Press; 2025. p. 3–19.

[10] Bayliss WM , Starling EH . The mechanism of pancreatic secretion. J Physiol. 1902;28:325–353. 16992627 10.1113/jphysiol.1902.sp000920 PMC1540572

[11] Ivy AC , Oldberg E . Hormone mechanism for gallbladder contraction and evacuation. Am J Physiol. 1928;86:599–613.

[12] Mutt V , Jorpes JE , Structure of porcine cholecystokinin‐pancreozymin. I. Cleavage with thrombin and with trypsin. Eur J Biochem. 1968;6:156–162. 5725809 10.1111/j.1432-1033.1968.tb00433.x

[13] Rehfeld JF . Cholecystokinin—from local gut hormone to ubiquitous messenger. Front Endocrinol. 2017;8:47. 10.3389/fendo.2017.00047 PMC5389988 28450850

[14] Rehfeld JF . Cholecystokinin—portrayal of an unfolding peptide messenger system. Peptides. 2025;186:171369. 39983917 10.1016/j.peptides.2025.171369

[15] Gregory H , Hardy PM , Jones DS , Kenner GW , Sheppard RC . The antral hormone gastrin. Structure of gastrin. Nature 1964;204:931–933. 14248711 10.1038/204931a0

[16] Anastasi A , Erspamer V , Exdean R . Isolation and amino acid sequence of caerulein, the active decapeptide of the skin of hyla caerulea. Arch Biochem Biophys. 1968;125:57–68. 5649531 10.1016/0003-9861(68)90638-3

[17] Anastasi A , Bertaccini G , Cei JM , De Caro G , Erspamer V , Impicciatore M . Structure and pharmacological actions of phyllocaerulein, a caerulein‐like nonapeptide: its occurrence in extracts of the skin of *Phyllomedusa sauvagei* and related *Phyllomedusa* species. Br J Pharmacol. 1969;37:198–206. 5824931 10.1111/j.1476-5381.1969.tb09538.x PMC1703762

[18] Johnsen AH , Rehfeld JF . Cionin: a disulfotyrosyl hybrid of cholecystokinin and gastrin from the neural ganglion of the protochordate *Ciona intestinalis* . J Biol Chem. 1990;265:3054–3058. 2303439

[19] Nachman RJ , Holman GM , Haddon WF , Ling N . Leucosulfakinin, a sulfated insect neuropeptide with homology to gastrin and cholecystokinin. Science 1986;234:71–73. 3749893 10.1126/science.3749893

[20] Nichols R , Schneuwly SA , Dixon JE . Identification and characterization of a drosophila homologue to the vertebrate neuropeptide cholecystokinin. J Biol Chem. 1988;263:12167–70. 2842322

[21] Johnsen AH . Phylogeny of the cholecystokinin/gastrin family. Front Neuroendocrinol. 1998;19:73–99. 9578981 10.1006/frne.1997.0163

[22] Baldwin GS , Patel O , Shulkes A . Evolution of gastrointestinal hormones: the cholecystokinin/gastrin family. Curr Opin Endocrinol Diabetes Obes. 2010;17:77–88. 19952740 10.1097/MED.0b013e328334e535

[23] Dupré D , Tostivint H . Evolution of the gastrin‐cholecystokinin gene family revealed by synteny analysis. Gen Comp Endocrinol. 2014;195:164–173. 24231682 10.1016/j.ygcen.2013.10.019

[24] Nässel DR , Wu SF . Evolutionary conserved roles of cholecystokinin signaling. In: Feinle‐Bisset C , Rehfeld JF , editors. *Cholecystokinin*—*from gallbladder to cognition and beyond* . Elsevier/Academic Press; 2025. p. 21–69.

[25] Deschenes RJ , Haun RS , Funckes CL , Dixon JE . A gene encoding rat cholecystokinin. Isolation, nucleotide sequence, and promoter activity. J Biol Chem. 1985;260:1280–1286. 2981840

[26] Takahashi Y , Kato K , Hayashizaki Y , Wakabayashi T , Ohtsuka E , Matsuki S , et al. Molecular cloning of the human cholecystokinin gene by use of a synthetic probe containing deoxyinosine. Proc Natl Acad Sci USA. 1985;82:1931–1935. 3856870 10.1073/pnas.82.7.1931 PMC397449

[27] Gubler U , Chua AO , Hoffman BJ , Collier KJ , Eng J . Cloned cDNA to cholecystokinin mRNA predicts an identical preprocholecystokinin in pig brain and gut. Proc Natl Acad Sci USA. 1984;81:4307–4310. 6205394 10.1073/pnas.81.14.4307 PMC345577

[28] Rehfeld JF , Bundgaard JR . Cell‐specific precursor processing. In: Rehfeld JF , Bundgaard JR , editors. Cellular peptide hormone synthesis and secretory pathways. Berlin, Heidelberg: Springer Verlag; 2010. p. 45–62.

[29] Dockray GJ . Immunochemical evidence of cholecystokinin‐like peptides in brain. Nature 1976;264:568–570. 63918 10.1038/264568a0

[30] Rehfeld JF . Immunochemical studies on cholecystokinin. II. Distribution and molecular heterogeneity in the central nervous system and small intestine of man and hog. J Biol Chem. 1978;253:4022–4030. 649618

[31] Goltermann NR , Rehfeld JF , Roigaard‐Petersen H . In vivo biosynthesis of cholecystokinin in rat cerebral cortex. J Biol Chem. 1980;255:6181–6185. 7391015

[32] Rehfeld JF , Hansen HF . Characterization of preprocholecystokinin products in the porcine cerebral cortex. Evidence of different processing pathways. J Biol Chem. 1986;261:5832–5840. 3700374

[33] Reeve JR , Eysselein V , Walsh JH , Ben‐Avram CM , Shively JE . New molecular forms of cholecystokinin. Microsequence analysis of forms previously characterized by chromatographic methods. J Biol Chem. 1986;261:16392–16397. 2430967

[34] Shively J , Reeve JR , Eysselein VE , Ben‐Avram C , Vigna SR , Walsh JH . CCK‐5: sequence analysis of a small cholecystokinin from canine brain and intestine. Am J Physiol. 1987;252:G272–G275. 3826354 10.1152/ajpgi.1987.252.2.G272

[35] Agersnap M , Rehfeld JF . Nonsulfated cholecystokinins in the small intestine of pigs and rats. Peptides 2015;71:121–127. 26206288 10.1016/j.peptides.2015.07.010

[36] Rehfeld JF . The endoproteolytic maturation of progastrin and procholecystokinin. J Mol Med. 2006;84:544–550. 16680481 10.1007/s00109-006-0055-3

[37] Rehfeld JF , Feinle‐Bisset C . The biogenesis and cell‐specific expression of cholecystokinin peptides. In: Feinle‐Bisset C , Rehfeld JF , editors. *Cholecystokinin*—*from gallbladder to cognition and beyond* . Elsevier/Academic Press; 2025. p. 71–85.

[38] Polak JM , Bloom SR , Rayford PL , Pearse AGE , Buchan AMJ , Thompson JC . Identification of cholecystokinin and secretin cells. Lancet 1975;2:1016–1017. 53500 10.1016/s0140-6736(75)90297-4

[39] Buffa R , Solcia E , Go VLW . Immunohistochemical identification of the cholecystokinin cell in the intestinal mucosa. Gastroenterology 1976;70:528–532. 767195

[40] Buchan AM , Polak JM , Solcia E , Capella C , Hudson D , Pearse AG . Election immunocytochemical evidence for the human intestinal I cell as the source of CCK. Gut 1978;19:403–407. 350727 10.1136/gut.19.5.403 PMC1412095

[41] Larsson LI , Rehfeld JF . Distribution of gastrin and CCK cells in the rat gastrointestinal tract. Histochemistry 1978;58:23–31. 365836 10.1007/BF00489946

[42] Fakhry J , Wang J , Martins P , Fothergill LJ , Hunne B , Prieur P , et al. Distribution and characterization of CCK enteroendocrine cells in the mouse small and large intestine. Cell Tissue Res. 2017;369:245–253. 28413860 10.1007/s00441-017-2612-1

[43] Gilliam‐Vigh H , Jorsal T , Rehfeld JF , Pedersen J , Poulsen SS , Vilsbøll T , et al. Expression of cholecystokinin and its receptors in the intestinal tract of type 2 diabetes patients and healthy controls. J Clin Endocrinol Metab. 2021;106:2164–2170. 34036343 10.1210/clinem/dgab367

[44] Larsson LI , Rehfeld JF . Localization and molecular heterogeneity of cholecystokinin in the central and peripheral nervous system. Brain Res. 1979;165:201–218. 369662 10.1016/0006-8993(79)90554-7

[45] Emson PC , Rehfeld JF , Rossor MN . Distribution of cholecystokinin‐like peptides in the human‐brain. J Neurochem. 1982;38:1177–1179. 7062038 10.1111/j.1471-4159.1982.tb05369.x

[46] Marley PD , Rehfeld JF , Emson PC . Distribution and chromatographic characterisation of gastrin and cholecystokinin in the rat central nervous system. J Neurochem. 1984;42:1523–1535. 6327906 10.1111/j.1471-4159.1984.tb12738.x

[47] Crawley JN . Comparative distribution of cholecystokinin and other neuropeptides. Why is this peptide different from all other peptides? Ann NY Acad Sci. 1985;448:1–8. 10.1111/j.1749-6632.1985.tb29900.x 3861115

[48] Hökfelt T , Barde S , Zhong W , He J . Cholecystokinin in the central and peripheral nervous system. In: Feinle‐Bisset C , Rehfeld JF , editors. *Cholecystokinin*—*from gallbladder to cognition and beyond* . Elsevier/Academic Press; 2025. p. 101–160.

[49] Schultzberg M , Hökfelt T , Nilsson G , Terenius L , Rehfeld JF , Brown M , et al. Distribution of peptide‐ and catecholamine‐containing neurons in the gastro‐intestinal tract of rat and guinea‐pig: immunohistochemical studies with antisera to substance P, vasoactive intestinal polypeptide, enkephalins, somatostatin, gastrin/cholecystokinin, neurotensin and dopamine beta‐hydroxylase. Neuroscience 1980;5:689–744. 6156425 10.1016/0306-4522(80)90166-9

[50] Rehfeld JF . Accumulation of nonamidated preprogastrin and preprocholecystokinin products in porcine pituitary corticotrophs. Evidence of post‐translational control of cell differentiation. J Biol Chem. 1986;261:5841–5847. 3700375

[51] Rehfeld JF . Preprocholecystokinin processing in the normal human anterior pituitary. Proc Natl Acad Sci USA. 1987;84:3019–3023. 3472248 10.1073/pnas.84.9.3019 PMC304792

[52] Laurberg P , Rehfeld JF . Cholecystokinin peptides as local modulators of thyroidal calcitonin secretion in the dog? J Endocrinol. 1987;115:77–82. 3668449 10.1677/joe.0.1150077

[53] Rehfeld JF , Johnsen AH , Ødum L , Bardram L , Schifter S , Scopsi L . Non‐sulphated cholecystokinin in human medullary thyroid carcinomas. J Endocrinol. 1990;124:501–506. 2332719 10.1677/joe.0.1240501

[54] Bardram L , Hilsted L , Rehfeld JF . Cholecystokinin, gastrin and their precursors in pheochromocytomas. Acta Endocrinol. 1989;120:479–484. 10.1530/acta.0.1200479 2718700

[55] Persson H , Ericsson A , Schalling M , Rehfeld JF , Hökfelt T . Detection of cholecystokinin in spermatogenic cells. Acta Physiol Scand. 1988;134:565–566. 3074624 10.1111/j.1748-1716.1998.tb08534.x

[56] Persson H , Rehfeld JF , Ericsson A , Schalling M , Pelto‐Huikko M , Hökfelt T . Transient expression of the cholecystokinin gene in male germ cells and accumulation of the peptide in the acrosomal granule: possible role of cholecystokinin in fertilization. Proc Natl Acad Sci USA. 1989;86:6166–6170. 2668956 10.1073/pnas.86.16.6166 PMC297798

[57] Goetze JP , Johnsen AH , Kistorp C , Gustafsson F , Johnbeck CB , Rehfeld JF . Cardiomyocyte expression and cell‐specific processing of procholecystokinin. J Biol Chem. 2015;290:6837–6843. 25627687 10.1074/jbc.M114.622670 PMC4358109

[58] Okahata H , Nishi Y , Muraki K , Sumii K , Miyachi Y , Usui T . Gastrin/cholecystokinin‐like immunoreactivity in human blood cells. Life Sci. 1985;36:369–373. 3965853 10.1016/0024-3205(85)90123-7

[59] Sacerdote P , Breda M , Barcellini W , Meroni PL , Panerai AE . Age‐related changes of beta‐endorphin and cholecystokinin in human and rat mononuclear cells. Peptides. 1991;12:1353–1356. 1815222 10.1016/0196-9781(91)90219-f

[60] Panza G , Monzani E , Sacerdote P , Penati G , Panerai AE . Beta‐endorphin, vasoactive intestinal peptide and cholecystokinin in peripheral blood mononuclear cells from healthy subjects and from drug‐free and haloperidol‐treated schizophrenic patients. Acta Psychiatr Scand. 1992; 85:207–210. 1561892 10.1111/j.1600-0447.1992.tb08596.x

[61] Rehfeld JF , Bundgaard JR , Hannibal J , Zhu X , Norrbom C , Steiner DF , et al. The cell‐specific pattern of cholecystokinin peptides in endocrine cells versus neurons is governed by the expression of prohormone convertases 1/3, 2, and 5/6. Endocrinology 2008;149:1600–1608. 18096669 10.1210/en.2007-0278 PMC2734493

[62] Rehfeld JF , Sun G , Christensen T , Hillingsø JG . The predominant cholecystokinin in human plasma and intestine is cholecystokinin‐33. J Clin Endocrinol Metab. 2001;86:251–258. 11232009 10.1210/jcem.86.1.7148

[63] Eysselein VE , Eberlein GA , Hesse WH , Singer MV , Goebell H , Reeve JR Jr . Cholecystokinin‐58 is the major circulating form of cholecystokinin in canine blood. J Biol Chem. 1987;262:214–217. 3793725

[64] Miller LJ . Biochemical characterization of receptors for the cholecystokinin family of hormones. In: Thompson JC , editor. Gastrointestinal endocrinology: receptors and post‐receptor mechanisms. New York, London, Tokyo: Academic Press; 1990. p. 81–93.

[65] Jensen RT , Huang SC , von Schrenck T , Wank SA , Gardner JD . Cholecystokinin receptor antagonists: ability to distinguish various classes of cholecystokinin receptors. In: Thompson JC , editor. Gastrointestinal endocrinology: receptors and post‐receptor mechanisms. New York, London, Tokyo: Academic Press; 1990. p. 95–113.

[66] Wank SA , Harkins R , Jensen RT , Shapira H , de Weerth A , Slattery T . Purification, molecular cloning, and functional expression of the cholecystokinin receptor from rat pancreas. Proc Natl Acad Sci USA. 1992;89:3125–3129. 1313582 10.1073/pnas.89.7.3125 PMC48817

[67] Kopin AS , Lee YM , McBride EW , Miller LJ , Lu M , Lin HY , et al. Expression cloning and characterization of the canine parietal cell gastrin receptor. Proc Natl Acad Sci USA. 1992;89:3605–3609. 1373504 10.1073/pnas.89.8.3605 PMC48917

[68] Wank SA . Cholecystokinin receptors. Am J Physiol. 1995;269:G628–G646. 7491953 10.1152/ajpgi.1995.269.5.G628

[69] Dufresne M , Seva C , Fourmy D . Cholecystokinin and gastrin receptors. Physiol Rev. 2006;86:805–847. 16816139 10.1152/physrev.00014.2005

[70] He L , Shi H , Zhang Ge , Peng Y , Ghosh A , Zhang M , et al. A novel CCK receptor GPR173 mediates potentiation of GABAergic inhibition. J Neurosci. 2023;43:2305–2325. 36813575 10.1523/JNEUROSCI.2035-22.2023 PMC10072296

[71] Henriksen FW , Worning H . The interaction of secretin and pancreozymin on the exocrine pancreatic secretion in dogs. Acta Physiol Scand. 1967;70:241–249.

[72] Wormsley KG . A comparison of the response to secretin, pancreozymin and a combination of these hormones, in man. Scand J Gastroenterol. 1969;4:411–417. 5351597

[73] Suarez‐Pinzon WL , Power RF , Yan Y , Wasserfall C , Atkinson M , Rabinovitch A . Combination therapy with glucagon‐like peptide‐1 and gastrin restores normoglycemia in diabetic NOD mice. Diabetes. 2008;57:3281–3288. 18835930 10.2337/db08-0688 PMC2584134

[74] Vana V , Lærke MK , Rehfeld JF , Arnold M , Dmytriyeva O , Langhans W , et al. Vagal afferent cholecystokinin receptor activation is required for glucagon‐like peptide‐1‐induced satiation. Diabetes Obes Metab. 2022;24:268–280. 34658116 10.1111/dom.14575

[75] Rehfeld JF . The CCKoma syndrome and its relation to the Zollinger–Ellison syndrome: a diagnostic challenge. Scand J Gastroenterol. 2024;59:533–542. 38299632 10.1080/00365521.2024.2308532

[76] Rehfeld JF , Feinle‐Bisset C . Reflections on cholecystokinin research: past, present and future. In: Feinle‐Bisset C , Rehfeld JF , editors. *Cholecystokinin*—*from gallbladder to cognition and beyond* . Elsevier/Academic Press; 2025. p. 597–600.

[77] Jorpes JE , Mutt V . On the bioassay of cholecystokinin preparations. In: Jorpes JE , Mutt V , editors. Secretin, cholecystokinin, pancreozymin and gastrin. Berlin, Heidelberg, New York: Springer Verlag; 1973. p. 140–144.

[78] Liddle RA , Goldfine ID , Rosen MS , Taplitz RA , Williams JA . Cholecystokinin bioactivity in human plasma. Molecular forms, responses to feeding, and relationship to gallbladder contraction. J Clin Invest. 1985;75:1144–1152. 2580857 10.1172/JCI111809 PMC425438

[79] Rehfeld JF . How to measure cholecystokinin in tissue, plasma and cerebrospinal fluid. Regul Peptides. 1998;78:31–39. 10.1016/s0167-0115(98)00133-5 9879744

[80] Rehfeld JF . Measurement of cholecystokinin in plasma with reference to nutrition related obesity studies. Nutr Res. 2020;76:1–8. 32109763 10.1016/j.nutres.2020.01.003

[81] Rehfeld JF . Immunochemical studies on cholecystokinin. I. Development of sequence‐specific radioimmunoassays for porcine triacontatriapeptide cholecystokinin. J Biol Chem. 1978;253:4016–4021. 565776

[82] Jansen J , Lamers C . Radioimmunoassay of cholecystokinin in human tissue and plasma. Clin Chim Acta. 1983;131:305–316. 6883724 10.1016/0009-8981(83)90100-6

[83] Chang TM , Chey WY . Radioimmunoassay of cholecystokinin. Dig Dis Sci. 1983;28:456–468. 6839908 10.1007/BF02430535

[84] Himeno S , Tarui S , Kanayama S , Kuroshima T , Shinomura Y , Hayashi C , et al. Plasma cholecystokinin responses after ingestion of liquid meal and intraduodenal infusion of fat, amino acids, or hydrochloric acid in man: analysis with region specific radioimmunoassay. Am J Gastroenterol. 1983;78:703–707. 6637957

[85] Becker HD , Werner M , Schafmayer A . Release of radioimmunologic cholecystokinin in human subjects. Am J Surg. 1984;147:124–129. 6691538 10.1016/0002-9610(84)90045-x

[86] Rehfeld JF , Accurate measurement of cholecystokinin in plasma. Clin Chem. 1998;44:991–1001. 9590372

[87] Foreman RE , Miedzybrodzka EL , Eiríksson FF , Thorsteinsdóttir M , Bannon C , Wheller R , et al. Optimized LC–MS/MS method for the detection of ppCCK(21–44): a surrogate to monitor human cholecystokinin secretion. J Proteome Res. 2023;22:2950–2958. 37591880 10.1021/acs.jproteome.3c00272 PMC10476265

[88] Cantor P , Rehfeld J . Cholecystokinin in pig plasma: release of components devoid of a bioactive COOH‐terminus. Am J Physiol. 1989;256:G53–G61. 2912150 10.1152/ajpgi.1989.256.1.G53

[89] Paloheimo LI , Rehfeld JF . Quantitation of procholecystokinin and its products in plasma by processing‐independent analysis. Clin Chim Acta. 1995;238:21–33. 7554293 10.1016/0009-8981(95)06055-i

[90] Albrechtsen NJW , Rehfeld JF . On premises and principles for measurement of gastrointestinal peptide hormones. Peptides 2021;141:170545. 33811948 10.1016/j.peptides.2021.170545

[91] Rehfeld JF , Goetze JP . Measurement of cholecystokinin in biological fluids. In: Feinle‐Bisset C , Rehfeld JF , editors. *Cholecystokinin*—*from gallbladder to cognition and beyond* . Elsevier/Academic Press; 2025. p. 423–440.

[92] Gibbs J , Young RC , Smith GP . Cholecystokinin elicits satiety in rats with open gastric fistulas. Nature 1973;245:323–325. 4586439 10.1038/245323a0

[93] Antin J , Gibbs J , Holt J , Young RC , Smith GP . Cholecystokinin elicits the complete behavioral sequence of satiety in rats. J Comp Physiol Psychol. 1975;89:784–790. 1176672 10.1037/h0077040

[94] Smith GP , Jerome C , Cushin BJ , Eterno R , Simansky KJ . Abdominal vagotomy blocks the satiety effect of cholecystokinin in the rat. Science 1981;213:1036–1037. 7268408 10.1126/science.7268408

[95] Browning K , Page AJ . Cholecystokinin and the gut‐brain axis. In: Feinle‐Bisset C , Rehfeld JF , editors. *Cholecystokinin*—*from gallbladder to cognition and beyond* . Elsevier/Academic Press; 2025. p. 219–251.

[96] Guilmeau S , Buyse M , Tsocas A , Laigneau JP , Bado A . Duodenal leptin stimulates cholecystokinin secretion: evidence of a positive leptin‐cholecystokinin feedback loop. Diabetes 2003;52:1664–1672. 12829630 10.2337/diabetes.52.7.1664

[97] Nolan LJ , Guss JL , Liddle RA , Pi‐Sunyer FX , Kissileff HR . Elevated plasma cholecystokinin and appetitive ratings after consumption of a liquid meal in humans. Nutrition 2003;19:553–557. 12781859 10.1016/s0899-9007(03)00039-x

[98] Sodowski K , Zwirska‐Korczala K , Kuka D , Kukla M , Budziszewska P , Czuba B , et al. Basal and postprandial gut peptides affecting food intake in lean and obese pregnant women. J Physiol Pharmacol. 2007;58(Suppl 1):37–52. 17443026

[99] Zwirska‐Korczala K , Konturek SJ , Sodowski M , Wylezol M , Kuka D , Sowa P , et al. Basal and postprandial plasma levels of PYY, ghrelin, cholecystokinin, gastrin and insulin in women with moderate and morbid obesity and metabolic syndrome. J Physiol Pharmacol. 2007;58(Suppl 1):13–35. 17443025

[100] Chearskul S , Delbridge E , Shulkes A , Proietto J , Kriketos A . Effect of weight loss and ketosis on postprandial cholecystokinin and free fatty acid concentrations. Am J Clin Nutr. 2008;87:1238–1246. 18469245 10.1093/ajcn/87.5.1238

[101] Jacobsen SH , Olesen SC , Dirksen C , Jørgensen NB , Bojsen‐Møller KN , Kielgast U , et al. Changes in gastrointestinal hormone responses, insulin sensitivity, and beta‐cell function within 2 weeks after gastric bypass in non‐diabetic subjects. Obes Surg. 2012;22:1084–1096. 22359255 10.1007/s11695-012-0621-4

[102] Mathus‐Vliegen EM , de Groot GH . Fasting and meal‐induced CCK and PP secretion following intragastric balloon treatment for obesity. Obes Surg. 2013;23:622–633. 23224567 10.1007/s11695-012-0834-6

[103] Dirksen C , Jørgensen NB , Bojsen‐Møller KN , Kielgast U , Jacobsen SH , Clausen TR , et al. Gut hormones, early dumping and resting energy expenditure in patients with good and poor weight loss response after Roux‐en‐Y gastric bypass. Int J Obes (Lond). 2013;37:1452–1459. 23419600 10.1038/ijo.2013.15

[104] Damgaard M , Graff J , Fuglsang S , Holst JJ , Rehfeld JF , Madsen JL . Effects of oleic acid and olive oil on gastric emptying, gut hormone secretion and appetite in lean and overweight or obese men. E‐Spen J. 2013;8:e8–e14.

[105] Meyer‐Gerspach AC , Häfliger S , Meili J , Doody A , Rehfeld JF , Drewe J , et al. Effect of L‐tryptophan and L‐leucine on gut hormone secretion, appetite feelings and gastric emptying rates in lean and non‐diabetic obese participants: a randomized, double‐blind, parallel‐group trial. PLoS ONE. 2016;11:e0166758. 27875537 10.1371/journal.pone.0166758 PMC5119776

[106] Svane MS , Jørgensen NB , Bojsen‐Møller KN , Dirksen C , Nielsen S , Kristiansen VB , et al. Peptide YY and glucagon‐like peptide‐1 contribute to decreased food intake after Roux‐en‐Y gastric bypass surgery. Int J Obes (Lond). 2016;40:1699–1706. 27434221 10.1038/ijo.2016.121

[107] Wölnerhanssen BK , Cajacob L , Keller N , Doody A , Rehfeld JF , Drewe J , et al. Gut hormone secretion, gastric emptying, and glycemic responses to erythritol and xylitol in lean and obese subjects. Am J Physiol. 2016;310:E1053–E1061. 10.1152/ajpendo.00037.2016 27117004

[108] Coutinho SR , With E , Rehfeld JF , Kulseng B , Truby H , Martins C . The impact of rate of weight loss on body composition and compensatory mechanisms during weight reduction: a randomized control trial. Clin Nutr. 2018;37:1154–1162. 28479016 10.1016/j.clnu.2017.04.008

[109] Coutinho SR , Rehfeld JF , Holst JJ , Kulseng B , Martins C . Impact of weight loss achieved through a multidisciplinary intervention on appetite in patients with severe obesity. Am J Physiol. 2018;315:E91–E98. 10.1152/ajpendo.00322.2017 29360396

[110] Dirksen C , Graff J , Fuglsang S , Rehfeld JF , Holst JJ , Madsen JL . Energy intake, gastrointestinal transit, and gut hormone release in response to oral triglycerides and fatty acids in men with and without severe obesity. Am J Physiol. 2019;316:G332–G337. 10.1152/ajpgi.00310.2018 30520691

[111] Svane MS , Bojsen‐Møller KN , Martinussen C , Dirksen C , Madsen JL , Reitelseder S , et al. Postprandial nutrient handling and gastrointestinal hormone secretion after Roux‐en‐Y gastric bypass vs sleeve gastrectomy. Gastroenterology 2019;156:1627–1641. 30742833 10.1053/j.gastro.2019.01.262

[112] DeBenedictis JN , Nymo S , Ollestad KH , Boyesen GA , Rehfeld JF , Holst JJ , et al. Changes in the homeostatic appetite system after weight loss reflect a normalization toward a lower body weight. J Clin Endocrinol Metab. 2020;105:e2538–e2546. 32301981 10.1210/clinem/dgaa202 PMC7250208

[113] Geary N , Asarian L , Graf G , Gobbi S , Tobler PN , Rehfeld JF , et al. Increased meal size but reduced meal‐stimulated plasma cholecystokinin concentrations in women with obesity. Endocrinology 2022;164:bqac192. 36423205 10.1210/endocr/bqac192

[114] Aukan MI , Rehfeld JF , Holst JJ , Martins C . Plasma concentration of gastrointestinal hormones and subjective appetite ratings after diet or bariatric surgery: 1‐year results from the DISGAP study. Int J Obes (Lond). 2025;49:306–314. 39572763 10.1038/s41366-024-01658-5

[115] de Krom M , Hendriks J , Hillebrand J , van Elburg A , Adan R . A polymorphism in the 3' untranslated region of the CCK gene is associated with anorexia nervosa in Dutch patients. Psychiatr Genet. 2006;16:239. 17106425 10.1097/01.ypg.0000242197.59020.2e

[116] Tamai H , Takemura J , Kobayashi N , Matsubayashi S , Matsukura S , Nakagawa T . Changes in plasma cholecystokinin concentrations after oral glucose tolerance test in anorexia nervosa before and after therapy. Metabolism 1993;42:581–584. 8492713 10.1016/0026-0495(93)90216-b

[117] Tomasik PJ , Sztefko K , Starzyk J . Cholecystokinin, glucose dependent insulinotropic peptide and glucagon‐like peptide 1 secretion in children with anorexia nervosa and simple obesity. J Pediatr Endocrinol Metab. 2004;17:1623–1631. 15645696 10.1515/jpem.2004.17.12.1623

[118] Harty RF , Pearson PH , Solomon TE , McGuigan JE . Cholecystokinin, vasoactive intestinal peptide and peptide histidine methionine responses to feeding in anorexia nervosa. Regul Pept. 1991;36:141–150. 1796180 10.1016/0167-0115(91)90202-r

[119] Heruc GA , Little TJ , Kohn MR , Madden S , Clarke SD , Horowitz M , et al. Effects of starvation and short‐term refeeding on gastric emptying and postprandial blood glucose regulation in adolescent girls with anorexia nervosa. Am J Physiol. 2018;315:E565–E573. 10.1152/ajpendo.00149.2018 29969316

[120] Cuntz U , Enck P , Frühauf E , Lehnert P , Riepl RL , Fichter MM , et al. Cholecystokinin revisited: CCK and the hunger trap in anorexia nervosa. PLoS ONE. 2013;8:e54457. 23349895 10.1371/journal.pone.0054457 PMC3547916

[121] Cox NJ , Morrison L , Ibrahim K , Robinson SM , Sayer AA , Roberts HC . New horizons in appetite and the anorexia of ageing. Age Ageing. 2020;49:526–534. 32043144 10.1093/ageing/afaa014

[122] Sturm K , MacIntosh CG , Parker BA , Wishart J , Horowitz M , Chapman IM . Appetite, food intake, and plasma concentrations of cholecystokinin, ghrelin, and other gastrointestinal hormones in undernourished older women and well‐nourished young and older women. J Clin Endocrinol Metab. 2003;88:3747–3755. 12915664 10.1210/jc.2002-021656

[123] MacIntosh CG , Morley JE , Wishart J , Morris H , Jansen J , Horowitz M , et al. Effect of exogenous cholecystokinin (CCK)‐8 on food intake and plasma CCK, leptin, and insulin concentrations in older and young adults: evidence for increased CCK activity as a cause of the anorexia of aging. J Clin Endocrinol Metab. 2001;86:5830–5837. 11739447 10.1210/jcem.86.12.8107

[124] Hannon‐Engel SL , Filin EE , Wolfe BE . CCK response in bulimia nervosa and following remission. Physiol Behav. 2013;122:56–61. 23988345 10.1016/j.physbeh.2013.08.014 PMC4395462

[125] Geracioti TD Jr , Liddle RA . Impaired cholecystokinin secretion in bulimia nervosa. N Engl J Med. 1988;319:683–688. 3412386 10.1056/NEJM198809153191105

[126] Devlin MJ , Walsh BT , Guss JL , Kissileff HR , Liddle RA , Petkova E . Postprandial cholecystokinin release and gastric emptying in patients with bulimia nervosa. Am J Clin Nutr. 1997;65:114–120. 8988922 10.1093/ajcn/65.1.114

[127] Munsch S , Biedert E , Meyer AH , Herpertz S , Beglinger C . CCK, ghrelin, and PYY responses in individuals with binge eating disorder before and after a cognitive behavioral treatment (CBT). Physiol Behav. 2009;97:14–20. 19419677 10.1016/j.physbeh.2009.01.015

[128] Smith KR , Moran TH . Cholecystokinin in obesity and other disorders of eating. In: Feinle‐Bisset C , Rehfeld JF , editors. Cholecystokinin—from gallbladder to cognition and beyond. Elsevier/Academic Press; 2025. p. 305–325.

[129] Tanday N , Irwin N , Flatt P . Islets effects of cholecystokinin in health, obesity and diabetes. In: Feinle‐Bisset C , Rehfeld JF , editors. Cholecystokinin—from gallbladder to cognition and beyond. Elsevier/Academic Press; 2025. p. 305–325.

[130] Rehfeld JF . Incretin physiology beyond glucagon‐like peptide 1 and glucose‐dependent insulinotropic polypeptide: cholecystokinin and gastrin peptides. Acta Physiol. 2011;201:405–411. 10.1111/j.1748-1716.2010.02235.x 21392266

[131] Rushakoff RA , Goldfine ID , Beccaria LJ , Mathur A , Brand RJ , Liddle RA . Reduced postprandial cholecystokinin (CCK) secretion in patients with noninsulin‐dependent diabetes mellitus: evidence for a role for CCK in regulating postprandial hyperglycemia. J Clin Endocrinol Metab. 1993;76:489–493. 8432795 10.1210/jcem.76.2.8432795

[132] Sonne DP , Rehfeld JF , Holst JJ , Vilsbøll T , Knop FK . Postprandial gallbladder emptying in patients with type 2 diabetes: potential implications for bile‐induced secretion of glucagon‐like peptide 1. Eur J Endocrinol. 2014;171:407–419. 24986531 10.1530/EJE-14-0309

[133] Rehfeld JF . Effect of gastrin and its C‐terminal tetrapeptide on insulin secretion in man. Acta Endocrinol. 1971;66:169–176. 10.1530/acta.0.0660169 5107421

[134] Larsson LI , Rehfeld JF . Peptidergic and adrenergic innervation of pancreatic ganglia. Scand J Gastroenterol. 1979;14:433–437. 384501

[135] Rehfeld JF , Larsson L‐I , Goltermann NR , Schwartz TW , Holst JJ , Jensen SL , et al. Neural regulation of pancreatic hormone secretion by the C‐terminal tetrapeptide of CCK. Nature 1980;284:33–38. 6101907 10.1038/284033a0

[136] Saillan‐Barreau C , Dufresne M , Clerc P , Sanchez D , Corominola H , Moriscot C , et al. Evidence for a functional role of the cholecystokinin‐B/gastrin receptor in the human fetal and adult pancreas. Diabetes 1999;48:2015–2021. 10512367 10.2337/diabetes.48.10.2015

[137] Reubi JC , Waser B , Gugger M , Friess H , Kleeff J , Kayed H , et al. Distribution of CCK_1_ and CCK_2_ receptors in normal and diseased human pancreatic tissue. Gastroenterology 2003;125:98–106. 12851875 10.1016/s0016-5085(03)00697-8

[138] Morisset J , Julien S , Lainé J . Localization of cholecystokinin receptor subtypes in the endocrine pancreas. J Histochem Cytochem. 2003;51:1501–1513. 14566022 10.1177/002215540305101110 PMC3957559

[139] Lavine JA , Raess PW , Stapleton DS , Rabaglia ME , Suhonen JI , Schueler KL , et al. Cholecystokinin is up‐regulated in obese mouse islets and expands beta‐cell mass by increasing beta‐cell survival. Endocrinology 2010;151:3577–3588. 20534724 10.1210/en.2010-0233 PMC2940525

[140] Lavine JA , Kibbe CR , Baan M , Sirinvaravong S , Umhoefer HM , Engler KA , et al. Cholecystokinin expression in the β‐cell leads to increased β‐cell area in aged mice and protects from streptozotocin‐induced diabetes and apoptosis. Am J Physiol. 2015;309:E819–E828. 10.1152/ajpendo.00159.2015 PMC4652070 26394663

[141] Linnemann AK , Neuman JC , Battiola TJ , Wisinski JA , Kimple ME , Davis DB . Glucagon‐like peptide‐1 regulates cholecystokinin production in β‐cells to protect from apoptosis. Mol Endocrinol. 2015;29:978–987. 25984632 10.1210/me.2015-1030 PMC4484781

[142] Irwin N , Frizelle P , Montgomery IA , Moffett RC , O'Harte FPM , Flatt PR . Beneficial effects of the novel cholecystokinin agonist (pGlu‐Gln)‐CCK‐8 in mouse models of obesity/diabetes. Diabetologia 2012;55:2747–2758. 22814764 10.1007/s00125-012-2654-6

[143] Irwin N , Montgomery IA , Moffett RC , Flatt PR . Chemical cholecystokinin receptor activation protects against obesity‐diabetes in high fat fed mice and has sustainable beneficial effects in genetic ob/ob mice. Biochem Pharmacol. 2013;85:81–91. 23085436 10.1016/j.bcp.2012.10.008

[144] Irwin N , Montgomery IA , O'Harte FP , Frizelle P , Flatt PR . Comparison of the independent and combined metabolic effects of subchronic modulation of CCK and GIP receptor action in obesity‐related diabetes. Int J Obes (Lond). 2013;37:1058–1063. 23164696 10.1038/ijo.2012.179

[145] Pathak V , Flatt PR , Irwin N . Cholecystokinin (CCK) and related adjunct peptide therapies for the treatment of obesity and type 2 diabetes. Peptides 2018;100:229–235. 29412823 10.1016/j.peptides.2017.09.007

[146] Nyborg NCB , Kirk RK , de Boer AS , Andersen DW , Bugge A , Wulff BS , et al. Cholecystokinin‐1 receptor agonist induced pathological findings in the exocrine pancreas of non‐human primates. Toxicol Appl Pharmacol. 2020:399:115035. 32422327 10.1016/j.taap.2020.115035

[147] Masclee AAM , Masclee GMC . Cholecystokinin and the gallbladder. In: Feinle‐Bisset C , Rehfeld JF , editors. Cholecystokinin—from gallbladder to cognition and beyond. Elsevier/Academic Press; 2025. p. 253–279.

[148] Hopman WP , Kerstens PJ , Jansen JB , Rosenbusch G , Lamers CB . Effect of graded physiologic doses of cholecystokinin on gallbladder contraction measured by ultrasonography. Determination of threshold, dose‐response relationships and comparison with intraduodenal bilirubin output. Gastroenterology 1985;89:1242–1247. 3902553 10.1016/0016-5085(85)90639-0

[149] Masclee AA , Jansen JB , Corstens FH , Lamers CB . Reversible gall bladder dysfunction in severe pancreatic insufficiency. Gut 1989;30:866–872. 2753411 10.1136/gut.30.6.866 PMC1434150

[150] Masclee AA , Jansen JB , Driessen WM , Geuskens LM , Lamers CB . Delayed plasma cholecystokinin and gallbladder responses to intestinal fat in patients with Billroth I and II gastrectomy. Surgery 1989;106:502–508. 2772825

[151] Masclee AA , Jansen JB , Driessen WM , Geuskens LM , Lamers CB . Effect of truncal vagotomy on cholecystokinin release, gallbladder contraction, and gallbladder sensitivity to cholecystokinin in humans. Gastroenterology 1990;98:1338–1344. 2323523 10.1016/0016-5085(90)90354-4

[152] de Boer SY , Masclee AA , Jebbink MC , Schipper J , Lemkes HH , Jansen JB , et al. Effect of acute hyperglycaemia on gall bladder contraction induced by cholecystokinin in humans. Gut 1993;34:1128–1132. 8174967 10.1136/gut.34.8.1128 PMC1374368

[153] Masclee AAM , Geuskens LM , Driessen WMM , Jansen JBM , Lamers C . Effect of aging on plasma cholecystokinin secretion and gallbladder emptying. AGE 1988;11:136–140.

[154] Geracioti TD , Nicholson WE , Orth DN , Ekhator NN , Loosen PT . Cholecystokinin in human cerebrospinal fluid: concentrations, dynamics, molecular forms and relationship to fasting and feeding in health, depression and alcoholism. Brain Res. 1993;629:260–268. 8111629 10.1016/0006-8993(93)91329-q

[155] Gjerris A , Rafaelsen OJ , Vendsborg P , Fahrenkrug J , Rehfeld JF . Vasoactive intestinal polypeptide decreased in cerebrospinal fluid (CSF) in atypical depression. Vasoactive intestinal polypeptide, cholecystokinin and gastrin in CSF in psychiatric disorders. J Affect Disord. 1984;7:325–337. 6241214 10.1016/0165-0327(84)90054-5

[156] Lotstra F , Verbanck PM , Gilles C , Mendlewicz J , Vanderhaeghen JJ . Reduced cholecystokinin levels in cerebrospinal fluid of parkinsonian and schizophrenic patients. Effect of ceruletide in schizophrenia. Ann NY Acad Sci. 1985;448:507–517. 3896098 10.1111/j.1749-6632.1985.tb29944.x

[157] Beinfeld MC , Garver DL . Concentration of cholecystokinin in cerebrospinal fluid is decreased in psychosis: relationship to symptoms and drug response. Prog Neuropsychopharmacol Biol Psychiatry. 1991;15:601–609. 1956989 10.1016/0278-5846(91)90050-b

[158] Bryld E , Zeeberg I , Gjerris A , Werdelin L , Rehfeld JF . Increased cerebrospinal fluid concentrations of C‐ but not N‐terminal cholecystokinin fragments in multiple sclerosis. Brain Res. 1987;409:364–366. 3580883 10.1016/0006-8993(87)90723-2

[159] Lo CM , Samuelson LC , Chambers JB , King A , Heiman J , Jandacek RJ , et al. Characterization of mice lacking the gene for cholecystokinin. Am J Physiol. 2008;294:R803–R810. 10.1152/ajpregu.00682.2007 18160529

[160] Lo CC , Black DD , Tso P . Cholecystokinin knockout mice: peripheral and central phenotypes. In: Feinle‐Bisset C , Rehfeld JF , editors. Cholecystokinin—from gallbladder to cognition and beyond. Elsevier/Academic Press; 2025. p. 461–475.

[161] Lacourse KA , Swanberg LJ , Gillespie PJ , Rehfeld JF , Saunders TL , Samuelson LC . Pancreatic function in CCK‐deficient mice: adaptation to dietary protein does not require CCK. Am J Physiol. 1999;276:G1302–G1309. 10330022 10.1152/ajpgi.1999.276.5.G1302

[162] Lo C‐M , Obici S , Dong HH , Haas M , Lou D , Kim DH , et al. Impaired insulin secretion and enhanced insulin sensitivity in cholecystokinin‐deficient mice. Diabetes 2011;60:2000–2007. 21602512 10.2337/db10-0789 PMC3121422

[163] Flood JF , Smith GE , Morley JE . Modulation of memory processing by cholecystokinin: dependence on the vagus nerve. Science 1987;236:832–834. 3576201 10.1126/science.3576201

[164] Itoh S , Takashima A , Maeda Y . Memory impairment induced by peripherally administered cholecystokinin A type receptor antagonists in rats. Drug Dev Res. 1992;26:89–99.

[165] Rehfeld JF . CCK and anxiety: an introduction. In: Dourish CT , Cooper SJ , Iversen SD , Iversen LL , editors. Multiple cholecystokinin receptors in man. Oxford: Oxford University Press; 1992. p. 117–120.

[166] Bradwejn J , Koszycki D , Meterissian G . Cholecystokinin‐tetrapeptide induces panic attacks in patients with panic disorder. Can J Psychiatry. 1990;35:83–85. 2180549 10.1177/070674379003500115

[167] Bradwejn J , Koszycki D . Cholecystokinin and panic disorder. In: Feinle‐Bisset C , Rehfeld JF , editors. Cholecystokinin—from gallbladder to cognition and beyond. Elsevier/Academic Press; 2025. p. 505–521.

[168] Horinouchi Y , Akiyoshi J , Nagata A , Matsushita H , Tsutsumi T , Isogawa K , et al. Reduced anxious behavior in mice lacking the CCK_2_ receptor gene. Eur Neuropsychopharmacol. 2004;14:157–161. 15013032 10.1016/S0924-977X(03)00103-2

[169] Reich N , Hölscher C . Cholecystokinin (CCK): a neuromodulator with therapeutic potential in Alzheimer's and Parkinson's disease. Front Neuroendocrinol. 2024;73:101122. 38346453 10.1016/j.yfrne.2024.101122

[170] Hökfelt T , Rehfeld JF , Skirboll L , Ivemark B , Goldstein M , Markey K . Evidence for coexistence of dopamine and CCK in meso‐limbic neurones. Nature 1980;285:476–478. 6105617 10.1038/285476a0

[171] Johnson LR . Trophic effects of gut peptides. In: Schultz SG, section editor, Makhlouf GM , volume editor. Handbook of physiology: the gastrointestinal system—neural and endocrine biology. 1989. p. 291–310.

[172] Gasslander T , Axelson J , Håkanson R , Ihse I , Lilja I , Rehfeld JF . Cholecystokinin is responsible for growth of the pancreas after pancreaticobiliary diversion in rats. Scand J Gastroenterol. 1990;25:1060–1065. 2263879 10.3109/00365529008997635

[173] Rehfeld JF , van Solinge WW . The tumor biology of gastrin and cholecystokinin. Adv Cancer Res. 1994;63:295–347. 8036989 10.1016/s0065-230x(08)60403-0

[174] Rehfeld JF . Cholecystokinin expression in tumors: biogenetic and diagnostic implications. Future Oncol. 2016;12:2135–2147. 27306028 10.2217/fon-2015-0053

[175] Madsen OD , Larsson LI , Rehfeld JF , Schwartz TW , Lernmark A , Labrecque AD , et al. Cloned cell lines from a transplantable islet cell tumor are heterogeneous and express cholecystokinin in addition to islet hormones. J Cell Biol. 1986;103:2025–2034. 2877997 10.1083/jcb.103.5.2025 PMC2114396

[176] Rehfeld JF , Federspiel B , Bardram L . A neuroendocrine tumor syndrome from cholecystokinin secretion. N Engl J Med. 2013;368:1165–1166. 23514309 10.1056/NEJMc1215137

[177] Rehfeld JF , Federspiel B , Agersnap M , Knigge U , Bardram L . The uncovering and characterization of a CCKoma syndrome in enteropancreatic neuroendocrine tumor patients. Scand J Gastroenterol. 2016;51:1172–1178. 27191542 10.1080/00365521.2016.1183706

[178] Rehfeld JF , Lindholm J , Andersen BN , Bardram L , Cantor P , Fenger M , et al. Pituitary tumors containing cholecystokinin. N Engl J Med. 1987;316:1244–1247. 3033502 10.1056/NEJM198705143162004

[179] Friedman JM , Vitale M , Maimon J , Israel MA , Horowitz ME , Schneider BS . Expression of the cholecystokinin gene in pediatric tumors. Proc Natl Acad Sci U S A. 1992;89:5819–5823. 1631063 10.1073/pnas.89.13.5819 PMC402109

[180] Reubi JC , Koefoed P , Hansen TVO , Stauffer E , Rauch D , Nielsen FC , et al. Procholecystokinin as marker of human Ewing sarcomas. Clin Cancer Res. 2004;10:5523–5530. 15328192 10.1158/1078-0432.CCR-1015-03

[181] Rehfeld JF , van Solinge WW , Tos M , Thomsen J . Gastrin, cholecystokinin and their precursors in acoustic neuromas. Brain Res. 1990;530:235–238. 1702342 10.1016/0006-8993(90)91288-r

[182] Camby I , Salmon I , Danguy A , Pasteels J‐L , Brotchi J , Martinez J , et al. Influence of gastrin on human astrocytic tumor cell proliferation. J Natl Cancer Inst. 1996;88:594–600. 8609660 10.1093/jnci/88.9.594

[183] Kaufmann R , Schafberg H , Zieger M , Henklein P , Nowak G . Protein kinase C is involved in cholecystokinin octapeptide‐induced proliferative action in rat glioma C6 cells. Neuropeptides 1998;32:185–189. 9639259 10.1016/s0143-4179(98)90036-1

[184] Oikonomou E , Buchfelder M , Adams EF . Cholecystokinin (CCK) and CCK receptor expression by human gliomas: evidence for an autocrine/paracrine stimulatory loop. Neuropeptides 2008;42:255–265. 18423848 10.1016/j.npep.2008.02.005

[185] Geijer T , Folkesson R , Rehfeld JF , Monstein HJ . Expression of the cholecystokinin gene in a human (small‐cell) lung carcinoma cell‐line. FEBS Lett. 1990;270:30–32. 1977617 10.1016/0014-5793(90)81227-f

[186] Ødum L , Rehfeld JF . Expression and processing of procholecystokinin in a rat medullary thyroid carcinoma cell line. Biochem J. 1990;271:31–36. 2222420 10.1042/bj2710031 PMC1149510

[187] Kurosawa M , Iijima S , Funakoshi A , Kawanami T , Miyasaka K , Bucinskaite V , et al. Choleccystokinin‐8 (CCK‐8) has no effect on heart rate in rats lacking CCK‐A receptors. Peptides 2001;22:1279–1284. 11457521 10.1016/s0196-9781(01)00452-1

[188] Leigh RS , Ruskoaho HJ , Kaynak BL . Cholecystokinin peptide signaling is rregulated by a TBX5‐MEF2 axis in the heart. Peptides 2021;136:170459. 33249116 10.1016/j.peptides.2020.170459

[189] Zhang X , Grosfeld A , Williams E , Vasiliauskas D , Barretto S , Smith L , et al. Fructose malabsorption induces cholecystokinin expression in the ileum and cecum by changing microbiota composition and metabolism. FASEB J. 2019;33:7126–7142. 30939042 10.1096/fj.201801526RR PMC6988857

